# Empirical Assessment of the Impact of Low-Cost Generic Programs on Adherence-Based Quality Measures

**DOI:** 10.3390/pharmacy5010015

**Published:** 2017-03-08

**Authors:** Nathan J. Pauly, Jeffery C. Talbert, Joshua D. Brown

**Affiliations:** 1 Department of Pharmacy Practice & Science, University of Kentucky College of Pharmacy, Lexington, 40508 KY, USA; nathan.pauly@uky.edu (N.J.P.); jeff.talbert@uky.edu (J.C.T.); 2 Department of Pharmaceutical Outcomes & Policy, University of Florida College of Pharmacy, Gainesville, 32610 FL, USA

**Keywords:** low-cost generic medications, quality measurement, adherence

## Abstract

In the United States, federally-funded health plans are mandated to measure the quality of care. Adherence-based medication quality metrics depend on completeness of administrative claims data for accurate measurement. Low-cost generic programs (LCGPs) cause medications fills to be missing from claims data as medications are not adjudicated through a patient’s insurance. This study sought to assess the magnitude of the impact of LCGPs on these quality measures. Data from the 2012–2013 Medical Expenditure Panel Survey (MEPS) were used. Medication fills for select medication classes were classified as LCGP fills and individuals were classified as never, sometimes, and always users of LCGPs. Individuals were classified based on insurance type (private, Medicare, Medicaid, dual-eligible). The proportion of days covered (PDC) was calculated for each medication class and the proportion of users with PDC ≥ 0.80 was reported as an observed metric for what would be calculated based on claims data and a true metric which included missing medication fills due to LCGPs. True measures of adherence were higher than the observed measures. The effect’s magnitude was highest for private insurance and for medication classes utilized more often through LCGPs. Thus, medication-based quality measures may be underestimated due to LCGPs.

## 1. Introduction

Low-cost generic drug programs (LCGPs) in the United States (U.S.) increase the affordability and accessibility of prescription medication [1]. LCGPs are unique to the U.S market as a “loss-leader” pricing strategy, i.e., retailers accept a loss on these cheap medications to bring in customers. LCGPs are available at 8 of the top 10 pharmacy chains (e.g., Wal-Mart, Walgreens) and provide many of the most commonly used generic medications at copayments of $4–$5 for 30-day supplies or $10–$12 for 90-day supplies [1,2,3]. These prices are generally lower than the insurance co-payment for the medications; thus, patients using these programs acquire the medications without the insurance company’s knowledge.

Our group recently assessed the prevalence and patient characteristics associated with LCGP use among those who are privately [4] and publicly (Medicare) [5] insured as well as in uninsured [6] and pediatric [7] populations. In each study, we analyzed which medications are most commonly purchased through LCGPs, the prevalence of LCGP use at the individual level, and the predictors of LCGP use in nationally representative samples. These studies indicate that there is a higher prevalence of LCGP use than has been estimated in previous literature. Using these programs, a claim may not be submitted through an individual’s insurance benefit; thus, medication use data can be missing from administrative claims data. In the U.S., administrative claims are widely used as a data source for health plans to assess quality of care, safety surveillance, and research [8,9]. 

Quality measurement is mandated by the federal government for publicly funded insurance programs including Medicaid and Medicare Advantage (Part C) and Part D plans. The measures are based on a standard set of measures**—**including several regarding pharmaceutical utilization [10,11]. Medications filled through LCGPs may go unobserved in claims data, leading to an underestimation of overall medication use and potentially a lower quality score. These scores are particularly important considering that they have been linked to plan enrollment and can impact quality-based reimbursement packages in a “pay-for-performance” healthcare environment [12,13]. 

This study sought to empirically assess the impact LCGPs may have on quality measurement for health insurance plans by identifying individuals and medications fills which may be unobserved due to LCGP use. For the purpose of this analysis, we calculated the quality metric for the proportion of days covered (PDC) of several drug classes that are assessed by quality measures and are available through LCGPs [14]. 

## 2. Materials and Methods 

### 2.1. Data Source

This study used data from the Medical Expenditure Panel Survey (MEPS) from the years 2012**–**2013. These years were chosen as they are the two most recent years for which MEPS data are available. MEPS is a nationally representative survey of individuals living in the United States that collects data regarding demographics and clinical conditions, as well as healthcare and pharmaceutical utilization. MEPS is a de-identified public use dataset supported by the Agency for Healthcare Research and Quality (AHRQ) that is intended for research purposes; therefore, it is exempt from IRB approval. Details related to the design and data collection processes of MEPS are detailed elsewhere [15]. 

### 2.2. Study Design and Population

A retrospective longitudinal study design was used to compare medication adherence that would be observed by health plans to that which may go unobserved due to use of LCGPs. Individuals were included in the study sample if they completed all five rounds of MEPS data collection, participated in the pharmacy survey, and reported having any public (Medicare, Medicaid, dual) or private insurance coverage. Subjects who were uninsured were excluded. Study subjects were required to have at least two fills of a medication in non-consecutive rounds for one of the following medication classes: angiotensin converting enzyme inhibitors (ACEi), beta-blockers, calcium channel blockers, HMG Co-A reductase inhibitors (statins), metformin, and sulfonylureas. These inclusion criteria were selected to be as similar as possible to the criteria used to calculate PDC for standard HEDIS measures [11,14]. 

### 2.3. LCGP Use

Four stipulations were used to define LCGP use [4,5,6,7]: (1) The total cost of the drug was paid out of pocket; (2) The cost of the drug exactly matched the cost of an LCGP drug as reported by pharmacies; (3) The medication was listed as an LCGP from a major chain pharmacy; and (4) Medications were dispensed for 30 or 90 days supplied of medications. Individuals were classified as always, sometimes, or never using LCGPs to fill medications for each medication class. 

### 2.4. Outcome Measures

PDC was calculated for each person during calendar years 2012 and 2013 for each medication class of interest by summing the total number of days supplied of medication, divided by 365 days. In cases where the days supplied variable was missing, the value was imputed based off mean days supply given the quantity of drug dispensed by National Drug Code [16]. 

Two distinct PDC measures were derived to demonstrate the effect of LCGP medications on quality measurement. The observed *PDC* only considers individuals and fills that would be observed in administrative claims data including all non-LCGP fills by individuals who sometimes or never used LCGPs. LCGP fills were excluded as these fills would not be observed in claims, thus having a potential detrimental impact on calculation of PDC as medication users are included in the denominator, but missing fills are absent from the calculation. The true *PDC* measure represents the PDC that a health plan would have derived had it observed LCGP fills for individuals who sometimes used LCGPs. PDC was not calculated for “always” users as they would never be observed in claims data. However, the implications of these LCGP users was discussed.

The frequency distribution of individuals classified as always, sometimes, and never LCGP users was calculated for each insurance type and was stratified by medication class. Mean PDC as well as the proportion of individuals with a PDC greater than or equal to 0.8 was calculated for each PDC measure and was stratified by medication class and insurance type. All data analyses were conducted using SAS 9.4 (SAS Institute, Cary, NC, USA).

## 3. Results

Table 1 shows the number of users for each medication class and the distribution by insurance type. A higher proportion of private insurance beneficiaries were classified as always or sometimes using LCGPs compared to those with public insurance. The greatest proportions of individuals classified as never using LCGPs were observed for the dual-eligible and Medicare populations. This difference is likely driven by higher deductibles and copayment amounts paid for prescription medications, with public insurance types typically paying much less and less incentive to use LCGPs due to cost.

Table 2 shows the results of the PDC calculations for each medication and insurance category including the overall and true measures of adherence. The mean PDC across measurements was marginally different for each comparison. For example, the observed PDC was 0.80 for ACEi users with private insurance and increased to 0.82 accounting for LCGP fills.

The impact of LCGPs on quality measures is shown by the proportion of people classified as having PDC ≥ 0.80 as this is the measure of interest for adherence-based quality measures. In the ACEi private insurance group, accounting for LCGP fills for those who sometimes use LCGPs, increased the proportion of individuals with PDC ≥ 0.80 by nearly 10%. The magnitude of this effect varied by insurance types and by medication class, driven by the proportion of fills attained for each medication through LCGPs. 

## 4. Discussion

The potential impact of LCGPs on quality measurement for health insurers has been acknowledged for some time [1]. However, this is the first known study to empirically demonstrate how quality measurement may be impacted by these programs. Prior studies have shown potential cost-savings to health plans if members use LCGPs, effectively offloading the burden of medication costs from the insurer to the pharmacy chain [17,18]. Despite the potential for cost-savings, these programs undermine quality measurement efforts by health plans and, ultimately, may impact the net revenue of these companies as payment and enrollment are increasingly being tied to a plan’s quality measures [12,13]. If health plans can ensure that all medication fills are recorded while their members continue to use these cash-payment systems, the potential for cost savings to both the patient and health plan may be substantial [17].

Adherence-focused quality metrics are based off of benchmarks for all health plans. For example, Medicare Advantage and Medicare Part D plans are assigned a 1-to-5 star ranking based on the proportion of members attaining a PDC ≥ 0.80 [19]. The cut points between star ratings are often differences of only a few percentage points, making even a small change in the proportion of members who are adherent potentially relevant to a plan’s rating [19]. Calculation of measures that define these star ratings is an imperfect process, and several of these shortcomings are present in this study design as well. Plans may under-calculate adherence for individuals who are taken off of medications or switched to medications in other classes. The present study design also under-calculated adherence for these individuals. Fortunately, since star ratings are relative measures, and every plan will likely under-calculate these metrics for a similar proportion of the population, this does not immediately impact the findings of this study nor the cut points for star ratings themselves. However, plans that can account for medication fills through cash payment systems like LCGPs could gain a competitive advantage and attain higher quality measures.

Quality measures considering beneficial medications will be underestimated due to LCGP use; resulting in lower plan ratings for these measures and incorrectly suggesting that a plan’s membership is receiving lower quality of care. It is in a health plan’s best interest to be able to accurately estimate the true metric in these cases and implement interventions which capture the utilization of these medications. Managed care organizations providing Part D and Medicare Advantage plans have acknowledged the issue of LCGPs as it pertains to quality measurement and should continue to look for ways to work directly with pharmacies to ensure reporting of LCGP medication fills [10]. Commercial solutions have been developed [20], but more research is needed to determine the impact of such interventions on both the patient’s out-of-pocket expenditures and the health plan’s total costs and quality measurement.

Although individuals who always filled medications through LCGPs were included in this study, these beneficiaries will not directly impact adherence rates calculated by health plans. Adherence-based quality metrics are only derived for individuals who fill multiple prescriptions for certain drugs in a given year. If all of an individual’s medication fills are through LCGPs then a health plan may never classify this person as a medication user and they will not contribute to the overall mean adherence of a health plan’s population. In this study, individuals who always used LCGPs had comparable adherence rates to those who sometimes or never used LCGPs. The failure to observe their medication use will not directly hurt the adherence calculation. However, failing to observe use of certain medications may harm other quality metrics calculated by health plans. For example, some quality metrics stipulate that certain drugs be prescribed after some adverse medical events—i.e., beta blockers post myocardial infarction (MI). If a beneficiary exclusively fills beta blockers through LCGPs this subsequent medication use will be unobserved and thus make health plans appear worse off. 

There are several ways to mitigate the potential for unobserved medication use due to LCGPs. If pharmacists file claims even when patients pay entirely out-of-pocket, then LCGP utilizations will appear in administrative claims data as zero paid claims. The problem with this solution is that pharmacists and patients do not currently have any incentive to file claims for customers paying out-of-pocket [1]. Pharmacists may be unaware of a patient’s insurance status or may find obtaining this information unnecessary especially if they believe that submitting a claim will result in a copay that exceeds the LCGP fee. While anecdotally this is a common occurrence, Medicare plans can consider the cost of an LCGP-purchased medication as the “usual and customary” prices, which can then be reimbursed at the typical level of the given plan (e.g., copay of $1 for a $4 30-day supply)**—**making it an even more affordable option [21]. A system-wide change (e.g., via mandate from the Centers for Medicare and Medicaid Services) is likely needed to dissuade missing information in claims data. However, policy makers need to be aware of the factors that drive the use of LCGPS**—**mainly accessibility and affordability of prescription drugs, and take this into account so that patients do not experience interruptions in care.

## 5. Conclusions 

LCGPs offer an affordable means to receive affordable generic medications and are being widely used in the U.S. Given the potential impact of LCGP use on quality measurement in health care systems, insurers should look for solutions that increase the reporting of LCGP medication use without interrupting the affordable access to medications which drives the demand of these programs.

## Figures and Tables

**Table 1:** Users of low-cost generic programs (LCGPs) by insurance status for each medication class.

	Insurance	Never Use LCGPs	Always Use LCGPs	Sometimes Use LCGPs
ACE inhibitors (*N* = 1861)	Private *N* (%)	386 (58.8)	119 (18.1)	152 (23.1)
	Medicaid *N* (%)	164 (84.5)	8 (4.1)	22 (11.3)
	Medicare *N* (%)	599 (74.3)	69 (8.6)	138 (17.1)
	Dual *N* (%)	181 (88.7)	9 (4.4)	14 (6.9)
Beta-blockers (*N* = 1847)	Private *N* (%)	341 (65.8)	78 (15.1)	99 (19.1)
	Medicaid *N* (%)	115 (81.0)	8 (5.6)	19 (13.4)
	Medicare *N* (%)	753 (78.8)	63 (6.6)	140 (14.6)
	Dual *N* (%)	215 (93.1)	3 (1.3)	13 (5.6)
Calcium channel blockers (*N* = 1207)	Private *N* (%)	239 (72.4)	35 (10.6)	56 (17.0)
	Medicaid *N* (%)	80 (92.0)	1 (1.2)	6 (6.9)
	Medicare *N* (%)	515 (86.6)	21 (3.5)	59 (9.9)
	Dual *N* (%)	190 (97.4)	0 (0)	5 (2.6)
Statins (*N* = 2714)	Private *N* (%)	669 (76.1)	65 (7.4)	145 (16.5)
	Medicaid *N* (%)	177 (92.2)	2 (1.0)	13 (6.8)
	Medicare *N* (%)	1139 (88.3)	34 (2.6)	117 (9.1)
	Dual *N* (%)	342 (96.9)	2 (0.6)	9 (2.6)
Metformin (*N* = 1030)	Private *N* (%)	213 (59.8)	61 (17.1)	82 (23.0)
	Medicaid *N* (%)	107 (83.6)	3 (2.3)	18 (14.1)
	Medicare *N* (%)	288 (72.9)	52 (13.2)	55 (13.9)
	Dual *N* (%)	142 (94.0)	2 (1.3)	7 (4.6)
Sulfonylureas (*N* = 473)	Private *N* (%)	81 (63.8)	15 (11.8)	31 (24.4)
	Medicaid *N* (%)	51 (85.0)	2 (3.3)	7 (11.7)
	Medicare *N* (%)	152 (71.7)	22 (10.4)	38 (17.9)
	Dual *N* (%)	70 (94.6)	1 (1.4)	3 (4.1)

**Table 2:** Proportion of days covered (PDC) for each medication class by insurance type based on low-cost generic program utilization.

Medication Class	Insurance	Observed ^a^	True ^b^
ACE inhibitors Mean (SD) % PDC ≥ 0.80	Private	0.80 (0.24) 54.3%	0.82 (0.22) 64.2%
	Medicaid	0.80 (0.23) 61.9%	0.82 (0.22) 65.5%
	Medicare	0.84 (0.22) 67.9%	0.85 (0.21) 70.1%
	Dual	0.87 (0.18) 76.9%	0.87 (0.18) 77.9
Beta-blockers Mean (SD) % PDC ≥ 0.80	Private	0.82 (0.23) 64.0%	0.83 (0.22) 66.8%
	Medicaid	0.81 (0.24) 66.4%	0.83 (0.23) 69.3%
	Medicare	0.85 (0.22) 70.7%	0.86 (0.21) 72.1%
	Dual	0.89 (0.17) 82.2%	0.90 (0.17) 82.6%
Calcium channel blockers Mean (SD) % PDC ≥ 0.80	Private	0.76 (0.26) 57.5%	0.79 (0.25) 61.3%
	Medicaid	0.79 (0.24) 61.7%	0.80 (0.24) 62.8%
	Medicare	0.84 (0.22) 69.7%	0.85 (0.22) 71.0%
	Dual	0.82 (0.23) 71.9%	0.83 (0.23) 72.3%
Statins Mean (SD) % PDC ≥ 0.80	Private	0.82 (0.23) 63.3%	0.83 (0.22) 66.1%
	Medicaid	0.82 (0.22) 66.7%	0.83 (0.21) 68.6%
	Medicare	0.85 (0.21) 70.0%	0.85 (0.21) 71.2%
	Dual	0.84 (0.21) 69.6%	0.85 (0.21) 70.3%
Metformin Mean (SD) % PDC ≥ 0.80	Private	0.79 (0.24) 57.9%	0.81 (0.23) 61.5%
	Medicaid	0.83 (0.22) 68.7%	0.83 (0.21) 70.9%
	Medicare	0.86 (0.21) 71.4%	0.86 (0.20) 73.4%
	Dual	0.88 (0.18) 76.4%	0.88 (0.18) 78.2%
Sulfonylureas Mean (SD) % PDC ≥ 0.80	Private	0.83 (0.23) 66.3%	0.83 (0.23) 68.4%
	Medicaid	0.79 (0.24) 61.3%	0.80 (0.24) 61.3%
	Medicare	0.83 (0.23) 67.0%	0.84 (0.22) 68.6%
	Dual	0.85 (0.21) 72.6%	0.86 (0.20) 73.8%

^a^ Observed: Includes all non-LCGP fills for individuals who never and sometimes use LCGPs. Includes individuals who never and sometimes use LCGPs for calculation of % PDC ≥ 0.80; ^b^ True: Includes all fills for individuals who never and sometimes use LCGPs. Denominator of patients is same as Observed.

## References

[1] Choudhry N.K., Shrank W.H. (2010). Four-dollar generics—Increased accessibility, impaired quality assurance. N. Engl. J. Med..

[2] Czechowski J.L., Tjia J., Triller D.M. (2010). Deeply discounted medications: Implications of generic prescription drug wars. J. Am. Pharm. Assoc..

[3] Rucker N.L. (2010). $4 generics: How low, how broad, and why patient engagement is priceless. J. Am. Pharm. Assoc..

[4] Pauly N.J., Brown J.D. (2015). Prevalence of Low-Cost Generic Program Use in a Nationally Representative Cohort of Privately Insured Adults. J. Manag. Care Spec. Pharm..

[5] Pauly N.J., Brown J.D., Talbert J.C. (2016). Low-Cost Generic Program Use by Medicare Beneficiaries: Implications for Medication Exposure Misclassification in Administrative Claims Data. J. Manag. Care Spec. Pharm..

[6] Brown J.D., Pauly N.J., Talbert J.C. (2016). The Prevalence and Predictors of Low-Cost Generic Program Use in a Nationally Representative Uninsured Population. Pharmacy.

[7] Pauly N.J., Talbert J.C., Brown J.D. (2015). The Prevalence and Predictors of Low-Cost Generic Program Use in the Pediatric Population. Drugs Real World Outcomes.

[8] Schneeweiss S., Avorn J. (2005). A review of uses of health care utilization databases for epidemiologic research on therapeutics. J. Clin. Epidemiol..

[9] Robb M.A., Racoosin J.A., Sherman R.E., Gross T.P., Ball R., Reichman M.E., Midthun K., Woodcock J. (2012). The US Food and Drug Administration’s Sentinel Initiative: expanding the horizons of medical product safety. Pharmacoepidemiol. Drug Saf..

[10] American Pharmacists Association and Academy of Managed Care Pharmacy (2014). Medicare star ratings: stakeholder proceedings on community pharmacy and managed care partnerships in quality. J. Am. Pharm. Assoc..

[11] Pharmacy Quality Alliance (PQA) PQA Performance Measures. http://pqaalliance.org/measures/default.asp.

[12] Reid R.O., Deb P., Howell B.L., Shrank W.H. (2013). Association between Medicare Advantage plan star ratings and enrollment. JAMA.

[13] Erickson S.C., Leslie R.S., Patel B.V. (2014). Is there an association between the high-risk medication star ratings and member experience CMS star ratings measures?. J. Manag. Care Pharm..

[14] National Committee for Quality Assurance (NCQA) HEDIS 2015. http://www.ncqa.org/HEDISQualityMeasurement/HEDISMeasures/HEDIS2015.aspx.

[15] Agency for Healthcare Research and Quality (AHRQ) Medical Expenduture Panel Survey. http://meps.ahrq.gov/mepsweb/about_meps/index_researcher.jsp.

[16] Davidoff A.J., Miller G.E., Sarpong E.M., Yang E., Brandt N., Fick D.M. (2015). Prevalence of potentially inappropriate medication use in older adults using the 2012 Beers criteria. J. Am. Geriatr. Soc..

[17] Zhang Y., Zhou L., Gellad W.F. (2011). Potential savings from greater use of $4 generic drugs. Arch. Intern. Med..

[18] Zhang Y., Gellad W.F., Zhou L., Lin Y.J., Lave J.R. (2012). Access to and use of $4 generic programs in Medicare. J. Gen. Intern. Med..

[19] Centers for Medicare and Medicaid Services Trends in Part C & D Star Rating Measure Cut Points. https://www.cms.gov/Medicare/Prescription-Drug-Coverage/PrescriptionDrugCovGenIn/Downloads/2014-Trends-in-Part-C-and-D-Star-Rating-Measure-Cut-Points.pdf.

[20] RelayHealth Intervetion MessagingRx (TM). http://www.relayhealth.com/solutions/payer-solutions/Intervention-MessagingRx.html.

[21] Centers for Medicare and Medicaid Services Prescription Drug Benefit Manual: Chapter 14 Coordination of Benefits. https://www.cms.gov/medicare/prescription-drug-coverage/prescriptiondrugcovcontra/partdmanuals.html.

